# Amyloid PET imaging in multiple sclerosis: an ^18^F-florbetaben study

**DOI:** 10.1186/s12883-015-0502-2

**Published:** 2015-11-25

**Authors:** Jordi A. Matías-Guiu, María Nieves Cabrera-Martín, Jorge Matías-Guiu, Celia Oreja-Guevara, Cristina Riola-Parada, Teresa Moreno-Ramos, Juan Arrazola, José Luis Carreras

**Affiliations:** Department of Neurology, Hospital Clínico San Carlos. San Carlos Institute for Health Research (IdISSC), Universidad Complutense de Madrid, Calle Profesor Martín Lagos, S/N, Madrid, 28040 Spain; Department of Nuclear Medicine, Hospital Clínico San Carlos. San Carlos Institute for Health Research (IdISSC), Universidad Complutense de Madrid, Calle Profesor Martín Lagos, S/N, Madrid, 28040 Spain; Department of Radiology, Hospital Clínico San Carlos. San Carlos Institute for Health Research (IdISSC), Universidad Complutense de Madrid, Calle Profesor Martín Lagos, S/N, Madrid, 28040 Spain

**Keywords:** Multiple sclerosis, Positron emission tomography, Amyloid, Myelin, White matter, Amyloid imaging

## Abstract

**Background:**

Positron emission tomography (PET) images with amyloid tracers show normal uptake in healthy white matter, which suggests that amyloid tracers are potentially useful for studying such white matter diseases as multiple sclerosis (MS).

**Methods:**

Twelve patients diagnosed with MS (5 with RRMS, 5 with SPMS, and 2 with PPMS) and 3 healthy controls underwent studies with MRI and ^18^F-florbetaben-PET imaging. Images were preprocessed using Statistical Parametric Mapping software. We analysed ^18^F-florbetaben uptake in demyelinating plaques (appearing as hyperintense lesions in FLAIR sequences), in normal-appearing white matter, and in grey matter.

**Results:**

Mean standardized uptake value relative to cerebellum was higher in normally appearing white matter (NAWM) (1.51 ± 0.12) than in damaged white matter (DWM) (1.24 ± 0.12; *P* = .002). Mean percentage of change between NAWM and DWM was −17.56 % ± 6.22 %. This percentage of change correlated negatively with EDSS scores (r = −0.61, *p* < .05) and with age (r = −0.83, *p* < 0.01). Progressive forms of MS showed a more pronounced reduction of the uptake in DWM in comparison to relapsing-remitting form.

**Conclusions:**

Uptake of ^18^F-florbetaben in damaged white matter is lower than that occurring in normally-appearing white matter. These findings indicate that amyloid tracers may be useful in studies of MS, although further research is needed to evaluate the utility of amyloid-PET in monitoring MS progression.

**Electronic supplementary material:**

The online version of this article (doi:10.1186/s12883-015-0502-2) contains supplementary material, which is available to authorized users.

## Background

Multiple sclerosis (MS) is an inflammatory neurodegenerative disease of the central nervous system in which characteristic demyelinating lesions appear in the white matter [1]. Magnetic resonance imaging (MRI) is the most widely used, most sensitive, and most specific technique for identifying white and grey matter lesions. In fact, this technique plays a crucial role that is specified by current diagnostic criteria [2, 3].

Positron emission tomography (PET) is a functional imaging technique in which radiotracers are used to study numerous biological processes. PET findings are an important biomarker in some neurological disorders [4] such as dementia [5, 6]. Recently, there has been a growing interest in exploring the potential applications of PET in MS studies [7, 8]. In this regard, several tracers are being studied, such as ligands of the 18kDA translocator protein as a microglial activation marker (ie. PK11195, PRB28), as well as ^11^C-acetate, ligands of monoamine oxidase B and I2-imidazoline receptor to study astrocyte metabolism and activation [9, 10].

In the past few years, researchers have developed several PET radiotracers that permit in vivo studies of amyloid-beta deposits. Important radiotracers include ^11^C-Pittsburgh compound B (^11^C-PiB) and ^18^F-labelled PET amyloid tracers such as ^18^F-florbetaben, ^18^F-florbetapir, and ^18^F-flutemetamol [11, 12]. Studies in healthy subjects have demonstrated amyloid tracer uptake by white matter, which suggests that the tracers may also be used in white matter studies. A recent study employing ^11^C-PiB in baboons and 2 patients with MS suggests that this tracer may be used as a marker of myelin loss and repair in demyelinating diseases [13]. However, experience with amyloid tracers in the study of white matter diseases is still limited [14, 15].

Our purpose is to study ^18^F-florbetaben uptake in damaged and in normal white matter in a series of patients with MS.

## Methods

### Study population

Our study was approved by the hospital ethics committee and meets the standards established by the Declaration of Helsinki. All patients signed informed consent forms. Written informed consent for publication of their clinical details and/or clinical images was obtained from the patients. We included 12 patients diagnosed with MS according to the 2010 revision of the McDonald criteria [2]: 5 patients had relapsing-remitting MS (RRMS), 5 had secondary-progressive MS (SPMS), and 2 had primary-progressive MS (PPMS). Patients’ mean age was 47.3 years (range, 41–61); 9 were female (75 %), and mean time since the onset was 13.2 years (range, 3.5–19). Mean Expanded Disability Status Scale (EDSS) score was 4.9 (range, 3.5–6.5) (Table 1). Patients had not suffered any relapse during the previous 6 months, and were not on active treatment with corticosteroids or interferon. No patient had gadolinium-enhancement on MRI brain lesions. We also included 3 healthy subjects with 48, 50 and 52 years (1 male, 2 females). These subjects were voluntary and did not have a family history of either MS or Alzheimer’s disease. In all subjects, neuropsychological assessment was performed, in order to confirm that cognitive deficits were related only to MS in the patient’s group, and to exclude cognitive impairment in the healthy control group.

**Table 1:** Demographic data and main variables for each patient

Patients	*Age*	*Sex*	*Type of MS*	*Progression time (years)* ^a^	*EDSS*	*Current treatment*	*WM lesion volume on FLAIR (mL)*	*DWM SUVRc*	*NAWM SUVRc*	*NAWM volume (mL)*	*Uptake changes in WM (%)*
1	42	F	RR	3.5	3.5	Azathioprine	24.13	1.14	1.36	173.00	−16.04
2	42	F	RR	14	4.0	Fingolimod	51.83	1.21	1.27	131.80	−4.51
3	43	F	RR	12	4.0	Fingolimod	10.01	1.45	1.67	193.11	−12.98
4	44	F	RR	15	4.0	Natalizumab	58.30	1.37	1.57	149.43	−12.61
5	47	F	RR	12	3.5	Fingolimod	52.06	1.20	1.46	163.85	−17.60
6	41	M	SP	19	5.0	Fingolimod	47.49	1.41	1.61	155.32	−12.41
7	46	M	SP	17	5.0	Glatiramer acetate	19.23	1.25	1.60	186.48	−22.10
8	47	F	SP	16	5.0	Fingolimod	34.06	1.18	1.57	182.44	−24.47
9	48	M	SP	14	6.0	Teriflunamide	40.0	1.28	1.54	170.71	−17.11
10	50	M	SP	11	6.5	Azathioprine	45.60	1.09	1.45	178.60	−24.62
11	50	F	PP	14	6.0	Glatiramer acetate	8.58	1.31	1.70	189.53	−22.63
12	61	F	PP	11	6.5	None	16.69	1.07	1.40	157.32	−23.71
C-1	48	F	-	-	-	-	-			185.20	**
C-2	50	M	-	-	-	-	-			185.60	**
C-3	52	F	-	-	-	-	-			185.00	**

*C* Healthy control, *DWM* damaged white matter, *EDSS* expanded disability status scale, *F* female, *M* male, *NAWM* normally-appearing white matter;

*PP* primary-progressive, *RR* relapsing-remitting, *SP* secondary-progressive, *SUVRc* standardized uptake value relative to cerebellum, *WM* white matter

^a^Time since onset is given in years from the first exacerbation or symptom

^a^In healthy controls, uptake change was estimated between total white matter and the mean of the uptake in the ROIs of DWM developed for the MS patients

### Image acquisition

All subjects underwent brain MRI and ^18^F-florbetaben-PET studies.

MRI imaging was performed using a 1.5 T scanner (Signa HDxt, GE Healthcare, Milwaukee, USA). The protocol consisted of the following sequences:

1. T1-weighted 3D fast spoiled gradient-echo (FSPGR) inversion recovery sequence (axial plane; repetition time [TR], 12 ms; echo time [TE], 2.3 ms; inversion time [TI], 400 ms; slice thickness, 3 mm; spacing, 0.0 mm; number of excitations [NEX], 1; matrix, 256 × 192; and field of view [FOV], 25 × 20 cm).
2. T2-weighted fluid-attenuated inversion recovery (FLAIR) sequence (axial and sagittal plane; TR, 9102 ms; TE, 121 ms; TI, 2250 ms; slice thickness, 4 mm; spacing, 0.4 mm; NEX, 1; matrix, 256 × 192; FOV, 24 cm).
3. T2-weighted double-echo fast spin-echo (FSE) sequence (axial plane; TR, 2620 ms; TE 15/90 ms; echo train length [ETL], 8; slice thickness, 3 mm; spacing, 0.0 mm; NEX, 2; matrix, 256 x 256; FOV, 22 cm).
4. T1-weighted post-contrast FSE sequence (TR, 640; TE, 11.8; ETL, 2; slice thickness, 3 mm; spacing, 0.0 mm; NEX, 2; matrix, 256 × 192; FOV, 22 cm). Gadoteric acid (Dotarem, Guerbet) was administered at a standard dose of 0.1 mmol/kg for each MRI study; T1-weighted post-contrast images were acquired 5 minutes after contrast injection.

PET tests were performed using a Siemens Biograph™ TruePoint™ PET-CT platform with lutetium oxyorthosilicate crystals and 6-slice CT integrated with a latest-generation PET scanner. ^18^F-florbetaben was administered intravenously at a mean dose of 300 MBq. Static images were taken 90 minutes after injection of the tracer. A second set of images was acquired at 120 minutes in 5 of 12 patients. Acquisition time was 15 minutes. The reconstruction was performed using the true X method with 2 iterations and 21 subsets. We used a 30 cm field of view and a Gaussian filter with full width at half maximum of 4 mm. All results are given for acquisition at 90 minutes, unless otherwise specified.

### Image preprocessing and analysis

Images were preprocessed using Statistical Parametric Mapping software version 8 (SPM8) (The Wellcome Trust Centre for Neuroimaging, Institute of Neurology, University College of London) [16]. Each patient’s T1-weighted MRI image was co-registered with the PET image using the normalized mutual information technique, with the T1-weighted image as the reference image. The T1-weighted MRI image was warped to Montreal Neurological Institute (MNI) space using the T1 template provided by SPM8 to define the warp parameters. The same warp parameters were subsequently applied to normalize co-registered PET images to MNI space. Likewise, T1-weighted images were segmented into grey matter, white matter, and cerebrospinal fluid probability maps. Lesions in T1-weighed images were previously filled using the lesion filling tool provided by Lesion Segmentation Tool (see below) to ensure an appropriate preprocessing of images [17].

We performed a region of interest (ROI) analysis using MarsBaR software and the Automated Anatomical Labeling (AAL) atlas [18]. The ROI corresponding to each patient’s total white matter (TWM) was generated based on the white matter probability map. The AAL atlas was used to delimit white matter in the frontal, limbic, parietal, temporal, occipital, and sublobar regions in both hemispheres. We used the intersection of regions in the atlas with regions obtained from each patient to define ROIs using the ImCalc function in SPM8.

**Fig. 1:**
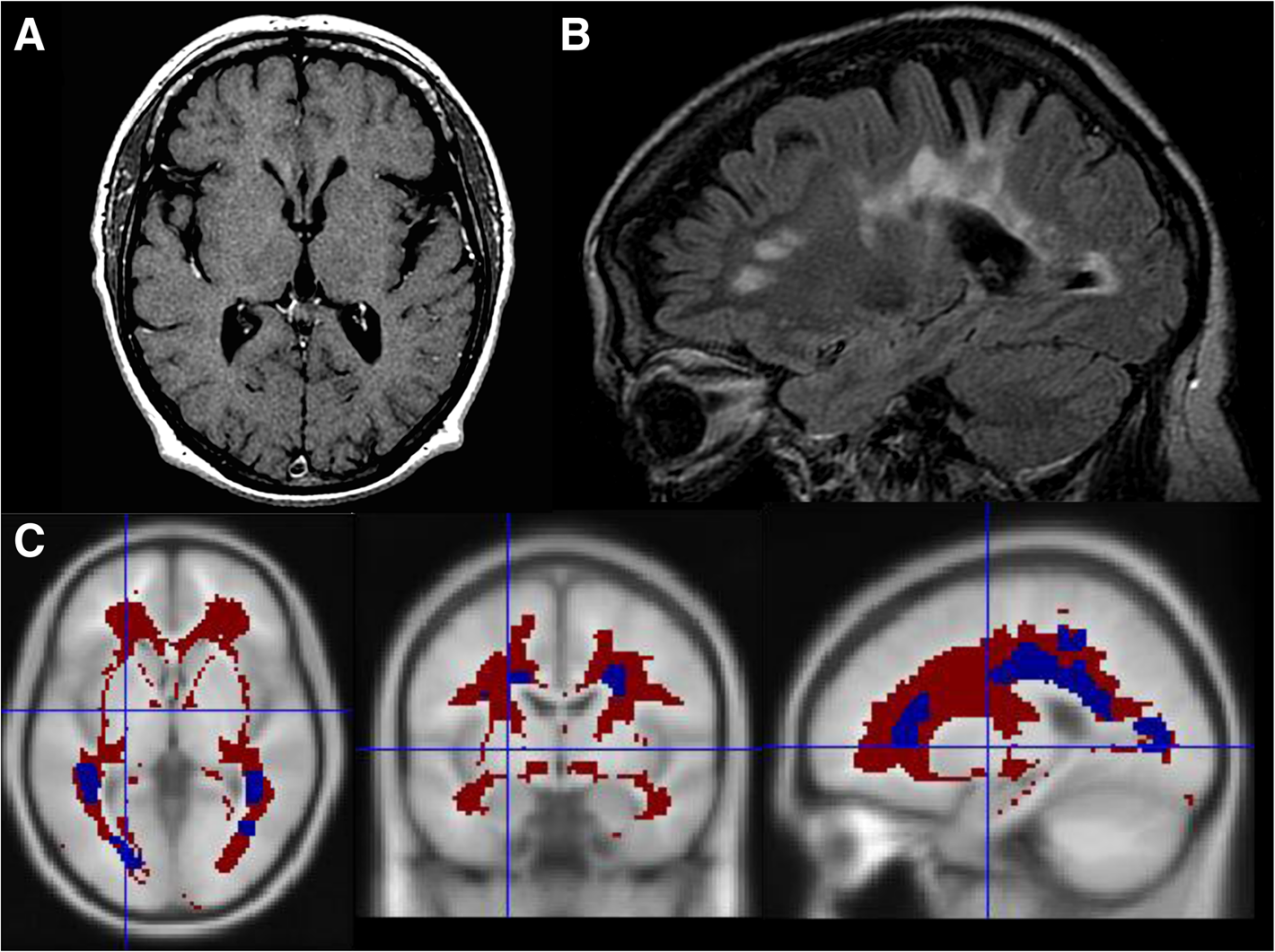
NAWM and DWM segmentation **a** T1-weighted sequence; **b** FLAIR sequence; **c** NAWM (red) and white matter lesion (blue) segmentation on a T1 template

The Lesion Segmentation Tool, developed by Schmidt et al. [19], was used to detect white matter lesions. This tool is based on an algorithm developed and validated for automatic segmentation of T2 hyperintense white matter lesions in MS. Segmentation is performed using a T2-weighted FLAIR imaging sequence and a 3D gradient echo T1-weighted sequence. A threshold K value of 0.3 was used [19]. ROIs of the white matter lesions were based on probability maps. We made sure that each of the ROIs obtained from the probability maps corresponded to a real lesion and eliminated any unnecessary ROIs. The adequate coregistration, normalization and agreement between lesion probability maps and lesions visually observed in MRI were checked by two of the researchers (JAM-G, MNC-M). Following this step, a single ROI for the damaged white matter (DWM) (i.e. T2-weighted FLAIR imaging lesions) was created. The ROI representing normal-appearing white matter (NAWM) was calculated by subtracting the DWM ROI from the TWM ROI (Fig. 1). Moreover, lesion probability maps were used to fill the T1-weighted images of each patient.

In healthy controls, imaging preprocessing was conducted in the same manner. TWM was estimated by the same procedure described above. In addition, the DWM ROIs developed in MS patients was applied to the healthy control, so that tracer uptake in the white matter located in the regions frequently damaged in MS could be evaluated.

Grey matter was evaluated using the following regions from the AAL atlas: anterior cingulate, posterior cingulate, middle frontal gyrus (orbital part), superior parietal gyrus, precuneus, superior temporal gyrus and superior occipital gyrus [18]. We used cerebellum and NAWM as the reference regions; results are presented as standardized uptake values (SUVRc relative to the cerebellum; SUVRwm relative to normally-appearing white matter). Whole cerebellum was used as a reference region after we checked that this region was not significantly impaired in the patients included in the study. In cases with any cerebellar lesion, this was masked and excluded from the analysis.

### Manual ROI delineation

In addition to the semi-automatic analysis described above, manual ROIs were generated by two raters (JAM-G, MNC-M) in a Siemens Leonardo workstation. Four white matters lesions (two periventricular and two juxtacortical) were delineated for each patient in T2-weighted FLAIR imaging sequence; and two ROIs in the normal appearing white matter. ROIs were used to calculate the SUV in the coregistered PET.

### Statistical analysis

Results are shown as means ± SD. We calculated the percentage of change between NAWM and DWM as follows: DWM uptake minus NAWM uptake, divided by NAWM uptake and multiplied by 100. The Mann–Whitney U test was used to compare means between two groups. The Wilcoxon test for paired samples was used to compare the same patient’s uptake in different regions (NAWM vs DWM, NAWM vs grey matter). Correlations were calculated using the Spearman correlation coefficient.

## Results

Mean SUV relative to the cerebellum (SUVRc) was higher in the NAWM (1.51 ± 0.12) than in the DWM (1.24 ± 0.12; *P* = .002). Mean percentage of change between NAWM and DWM was −17.56 % ± 6.2 %. Table 1 shows the SUVRc for each of the patients. The lower uptake in the white matter lesions was also observed in the manual delineated ROIs (Table 2), in which mean SUV in white matter lesions was 1.25 ± 0.21 and in normal appearing white matter was 1.76 ± 0.28 (*P =* .002). The RR group had a lower percentage of change between NAWM and DWM (−12.74 ± 5.05 vs. -21.00 ± 4.5, *P* = .04, respectively) and a lower EDSS score (3.80 ± 2.73 vs. 5.71 ± 0.69, *P* < .01) in comparison to the progressive forms of MS. However, no significant differences were obtained between both groups regarding age (43.60 ± 2.0 vs. 49.00 ± 6.11, *P* = .07) and total lesion volume in T2-weighted MRI (39.47 ± 20.85 vs. 30.25 ± 15.37, *P* = .26). In comparison to healthy controls, SUVRc in NAWM was lower in MS than in the total white matter in healthy controls (1.51 ± 0.12 vs. 1.74 ± 0.07, *P* = .01). This finding was also observed with manually delineated ROIs (SUV 1.76 ± 0.28 in MS vs. 2.36 ± 0.07 in healthy controls, *P* = .009).

**Table 2:** Standardized uptake value in ROIs manually delineated

Patients	*PV-1*	*PV-2*	*JC-1*	*JC-2*	*NAWM*	*NAWM*
1	1.28	0.86	1.07	0.96	1.58	1.56
2	0.83	0.93	0.93	0.94	1.41	1.27
3	1.68	1.52	1.51	1.35	2.09	2.10
4	1.38	1.40	0.78	1.61	1.61	1.86
5	1.47	1.50	1.57	2.02	2.12	2.28
6	1.27	1.04	1.66	1.75	2.07	2.27
7	0.90	1.54	1.57	1.61	1.77	1.78
8	1.30	1.47	0.82	1.17	1.76	2.13
9	1.11	1.12	0.91	1.01	1.42	1.49
10	1.20	0.97	1.32	0.90	1.70	1.57
11	1.29	1.52	0.96	1.10	1.78	1.69
12	1.12	1.23	1.28	1.31	1.52	1.60
Control 1	-	-	-	-	2.35	2.25
Control 2	-	-	-	-	2.30	2.39
Control 3	-	-	-	-	2.37	2.51

*PV* periventricular lesion, *JC* juxtacortical lesion, *NAWM* normal appearing white matter

Mean SUVRc in MS patients for grey matter regions was 0.93 ± 0.12 in the middle frontal gyrus (orbital part), 1.18 ± 0.11 in the anterior cingulate, 1.26 ± 0.12 in the posterior cingulate, 1.17 ± 0.05 in the precuneus, 0.98 ± 0.10 in the superior parietal gyrus, and 0.96 ± 0.08 in the superior temporal gyrus. Using white matter as the reference region, SUVRwm was 0.61 ± 0.09 in the middle frontal gyrus (orbital part), 0.78 ± 0.04 in the anterior cingulate, 0.83 ± 0.05 in the posterior cingulate, 0.77 ± 0.06 in the precuneus, 0.64 ± 0.07 in the superior parietal gyrus, and 0.63 ± 0.04 in the superior temporal gyrus. The above demonstrates a lower uptake by the grey matter than by the white matter (results for each patient are shown in Additional file 1: Table S1).

The percentage of change between NAWM and DWM was negatively correlated with EDSS (*r* = −0.61, *P* < .05) and age (*r* = −0.83, *P* < .001). No significant correlations were found with the years of evolution of the disease (*r* = 0.25, *P* > .05) and total lesion volume on T2-weighted MRI (*r* = −0.36, P > .05). We estimated a linear regression model to calculate the semi-partial correlations (*sr*) between the percentage of change and the variables age (*sr* = −0.339), MS form (*sr* = −0.455), years of duration of the illness (*sr* = 0.262), total lesion volume (*sr* = 0.038) and EDSS (*sr* = 0.264). However, all semi-partial correlations were not were not statistically significant (*P* > .05), with a trend in the MS form (*P* = .063).

We also assessed potential differences in region discrimination for images taken at a later point in time (120 minutes after tracer injection) in 5 patients. In this subgroup, SUVRc was 1.40 ± 0.10 for NAWM and 1.15 ± 0.08 for DWM in the first series of images at 90 minutes (*P* = 0.043); in the second series of images, mean SUVRc was 1.41 ± 0.13 for NAWM and 1.20 ± 0.07 for DWM (*P* = 0.043). The percentage of change between DWM and NAWM was −17.19 ± 8.05 % and −14.34 % ± 5.88 %, respectively. Correlation between the percentage of change and EDSS was the same at 90 and 120 minutes (*r* = −0.87, P > .05). The mean area under the curve for discrimination between the evaluated grey matter regions and NAWM was 0.84 for the first acquisition and 0.86 for the second.

## Discussion

**Fig. 2:**
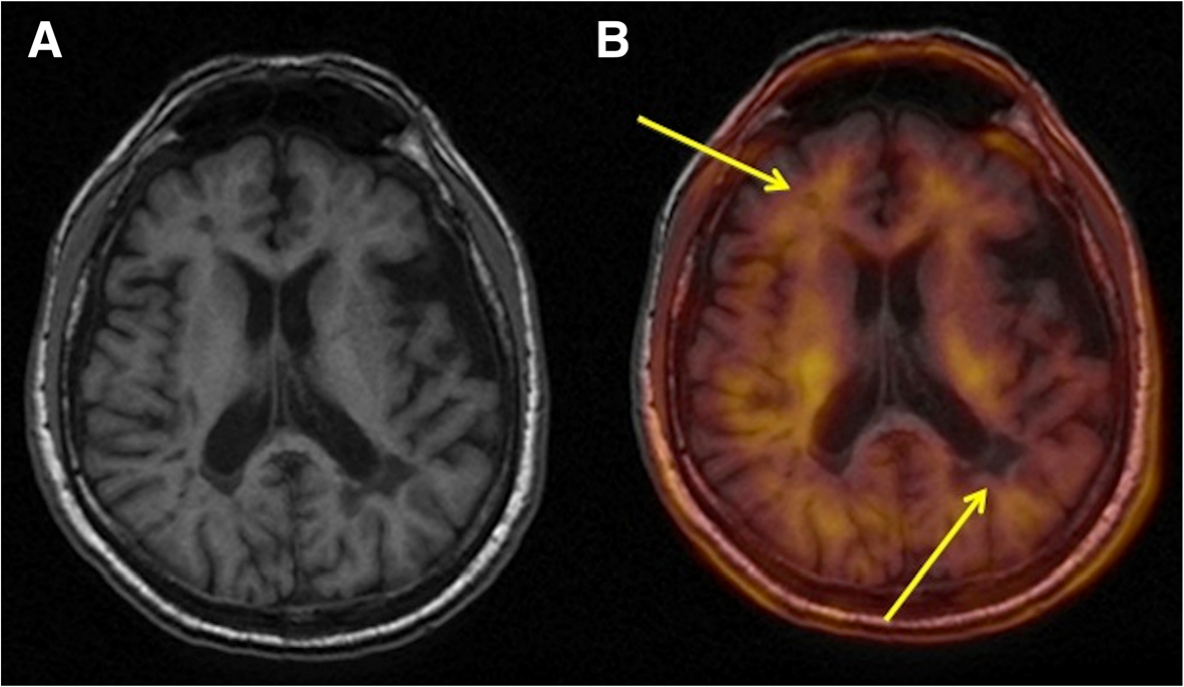
Co-registered MRI and ^18^F-florbetaben-PET images. **a** T1-weighted MRI. **b** Co-registered MRI and ^18^F-florbetaben-PET image. Arrows show lesions and areas of low uptake detected using PET

Our study shows that ^18^F-florbetaben uptake in white matter lesions in patients with MS is lower than in NAWM. This finding was observed in all patients in the study and was true of total DWM and of each of the individual lesions (Fig. 2). To the best of our knowledge, this is the first study using ^18^F-labelled amyloid tracers to investigate MS. Our findings are similar to those reported by Stankoff et al. in a study of 2 patients with MS who were analysed with PiB-PET. These authors observed lower uptake in white matter lesions in T1-weighted images [13].

Uptake of tracer in demyelinating lesions was 17.56 % lower than in NAWM. This percentage is similar to that reported in a previous study with PiB-PET [20] (Table 3). Furthermore, patients with progressive forms of MS and those with higher EDSS score displayed a more marked reduction in uptake in DWM. In line with the previous hypothesis that amyloid tracer fixation may be a marker of demyelination/remyelination, the more marked reduction in uptake observed in PP and SP MS may be associated with the reduced remyelination present in progressive forms of the disease [21]. In the same way, uptake reduction in DWM correlated with age. This might also be explained by the decrease of remyelination capacity with increasing age [22]. These findings support the notion that amyloid tracers, and ^18^F-florbetaben in particular, serve as markers of the extent of white matter demyelination. This suggests that amyloid tracers may indicate the state of myelin in the central nervous system since they experience non-specific uptake by white matter [13]. In this regard, several studies have also observed lower PiB uptake in elderly patients with white matter lesions [23, 24]. Although semi-partial correlations were not statistically significant, this might be explained by the small sample size that limits the use of the regression analysis.

**Table 3:** Studies of MS using amyloid-PET.

	*Number of cases*	*Type of MS*	*Amyloid tracer*	*Results*
Stankoff et al., 2011 [11]	2	RR	^11^C-PiB	Decreased focal PiB uptake in T1-weighted lesions
Bodini et al., 2013 [20]	12	Not specified	^11^C-PiB	Reduced uptake in white matter lesions
Present study	12	5 RR, 5 SP, 2 PP	^18^F-florbetaben	Reduced uptake in white matter lesions

Another interesting result is the observation of a lower uptake in NAWM in MS patients in comparison to healthy subjects. The concept of normal-appearing white matter is a matter of debate. MRI techniques, such as diffusion tension imaging, PET studies with radioligands of activated microglia, and pathological studies have demonstrated abnormalities in NAWM [25, 26]. The finding of a reduced 18 F-florbetaben uptake in NAWM might support the role of amyloid ligands in the assessment of the integrity of white matter. However, further studies specifically designed to address this issue are necessary to be able to draw definitive conclusions.

Regarding image acquisition time, uptake differences between DWM and NAWM at 120 minutes after tracer injection were slightly less marked than they had been at 90 minutes. Discrimination between NAWM and grey matter was very similar at 90 and 120 minutes. Amyloid tracer clearance is slower from white matter than from grey matter, which poses the question of what acquisition time is best and whether later acquisition times might be suitable for white matter studies [24]. Our findings do not seem to show later acquisition times to be suitable for distinguishing between NAWM and DWM or between white matter and grey matter. The fact that MS patients show low amyloid tracer uptake in grey matter probably indicates that discriminating between white matter and grey matter is unlikely to pose problems. This finding may be useful in other circumstances (for example, studying such age-related disorders as Alzheimer disease with associated white matter disease). However, further research into this specific subject is needed [23].

Our study has several limitations. Firstly, it is a preliminary study with a small sample size in which most of the patients presented high lesion load. Furthermore, acquired PET images were static and we were therefore unable to conduct a tracer kinetic study to analyse tracer behaviour in white matter over time. This approach could provide a better understanding of the role of amyloid PET in white matter diseases and possible modifications of the protocol of acquisition. However, the use of a static protocol should not affect the main objectives and results of the study regarding the reduction of PET signal in white matter lesions. One of the difficulties encountered using static acquisition is that it does not provide a true quantification; in fact, measurements are semi-quantitative and referenced to a region. Choosing this region of reference may affect the results. We have attempted to minimize this limitation by using two reference regions (cerebellum and white matter) and two methods of analysis (semi-automatic and manual measurements), in which the results have been reproduced.

## Conclusions

Our study shows reduced ^18^F-florbetaben uptake in white matter demyelinating lesions. These findings confirm that ^18^F-florbetaben is a marker of white matter damage in MS and support earlier evidence suggesting that amyloid tracers can be used to study white matter disease [13, 15, 22, 23]. Our study suggests that ^18^F-florbetaben may be useful in MS; however, longitudinal follow-up studies and larger samples are needed to increase our understanding of the information provided by amyloid-PET scans in patients with MS.

## Additional file

Additional file 1: Table S1. SUVRc and SUVRwm for grey matter regions (DOCX 94 kb)
