# Supplementary material for: Amyloid PET imaging in multiple sclerosis: an 18F-florbetaben study
Source: BMC Neurol. 2015 Nov 25;15:243. doi: 10.1186/s12883-015-0502-2 (PMC4660647; doi:10.1186/s12883-015-0502-2)
Supplement: Additional file 1: Table S1. — SUVRc and SUVRwm for grey matter regions (DOCX 94 kb) [file 12883_2015_502_MOESM1_ESM.docx]

|  | | Table 1. SUVRc and SUVRwm for grey matter regions | | | | | | | | | | | | |  | |  | |
| --- | --- | --- | --- | --- | --- | --- | --- | --- | --- | --- | --- | --- | --- | --- | --- | --- | --- | --- |
| Patients |  | | | | *SUVRc* | | | | | *SUVRwm* | | | | | | | | |
|  | MF | | AC | | PC | Precun | SP | ST | SO | MF | AC | PC | Precun | SP | | ST | | SO |
| 1 | .85 | | 1.07 | | 1.17 | 1.21 | 1.01 | .89 | 1.13 | .62 | .79 | .86 | .89 | .75 | | .66 | | .83 |
| 2 | .76 | | .93 | | 1.07 | 1.04 | .73 | .75 | .86 | .60 | .73 | .85 | .82 | .58 | | .59 | | .67 |
| 3 | .81 | | 1.27 | | 1.42 | 1.23 | .97 | 1.03 | 1.15 | .48 | .76 | .85 | .74 | .58 | | .62 | | .69 |
| 4 | 1.04 | | 1.26 | | 1.42 | 1.21 | 1.03 | .98 | 1.01 | .66 | .80 | .91 | .77 | .66 | | .63 | | .64 |
| 5 | 1.02 | | 1.23 | | 1.37 | 1.22 | 1.07 | 1.01 | 1.08 | .70 | .84 | .94 | .83 | .73 | | .69 | | .74 |
| 6 | .91 | | 1.24 | | 1.32 | 1.17 | .89 | .98 | .98 | .56 | .77 | .82 | .73 | .55 | | .61 | | .61 |
| 7 | .98 | | 1.24 | | 1.19 | 1.13 | 1.02 | 1.03 | 1.09 | .61 | .77 | .74 | .71 | .64 | | .64 | | .68 |
| 8 | .95 | | 1.35 | | 1.33 | 1.15 | .93 | 1.00 | 1.07 | .60 | .86 | .85 | .73 | .59 | | .64 | | .68 |
| 9 | 1.07 | | 1.20 | | 1.30 | 1.20 | 1.03 | 1.00 | 1.16 | .70 | .78 | .85 | .78 | .67 | | .65 | | .76 |
| 10 | .75 | | 1.08 | | 1.14 | 1.12 | .89 | .88 | 1.12 | .52 | .74 | .79 | .77 | .61 | | .61 | | .77 |
| 11 | .91 | | 1.19 | | 1.36 | 1.15 | 1.06 | .99 | 1.21 | .53 | .70 | .80 | .68 | .63 | | .59 | | .72 |
| 12 | 1.13 | | 1.17 | | 1.14 | 1.25 | 1.12 | 1.02 | 1.13 | .81 | .84 | .81 | .89 | .80 | | .73 | | .81 |
| C-1 | 1.06 | | 1.24 | | 1.66 | 1.35 | 1.05 | 1.13 | 1.21 | .63 | .67 | .98 | .80 | .62 | | .67 | | .72 |
| C-2 | 1.05 | | 1.32 | | 1.37 | 1.29 | .87 | .81 | .81 | .61 | .47 | .79 | .75 | .51 | | .47 | | .47 |
| C-3 | 1.10 | | 1.31 | | 1.70 | 1.38 | 1.11 | 1.14 | 1.07 | .60 | .62 | .93 | .75 | .61 | | .62 | | .59 |
|  | |  | | ***MF: middle frontal (orbital part); AC: anterior cingulate; PC: posterior cingulate; Precun: Precuneus; SP: superior parietal; ST: superior temporal gyrus; SO: superior occipital.*** | | | | | | | | | | | | | | |
